# Privacy-preserving computation in the post-quantum era

**DOI:** 10.1093/nsr/nwab115

**Published:** 2021-07-07

**Authors:** Yu Yu, Xiang Xie

**Affiliations:** The Department of Computer Science and Engineering, Shanghai Jiao Tong University, China; Shanghai Qi Zhi Institute, China; Shanghai Key Laboratory of Privacy-Preserving Computation, China; Shanghai Key Laboratory of Privacy-Preserving Computation, China

## Abstract

This perspectives article surveys the most promising privacy-preserving cryptographic technologies including secure multiparty computation, zero-knowledge proofs and fully homomorphic encryption, and their various real-world applications.

Privacy-preserving computation realizes joint computation among multiple mutually distrustful participants while preserving the privacy of the computation, which has a wide range of applications in the data industry. In the context of cryptography, privacy-preserving technologies refer to those that involve one or more of the following: multiparty computation (MPC), zero-knowledge proof (ZKP) and fully homomorphic encryption (FHE). We introduce these technologies below with machine learning applications as concrete examples.

MPC was first introduced by Yao [1] in the 1980s, where multiple parties, say *P*_1_, . . . , *P*_*n*_, jointly compute a public function on their private inputs *x*_1_, . . . , *x*_*n*_ in such a privacy-preserving way that nothing beyond the output *f*(*x*_1_, . . . , *x*_*n*_) is revealed to the participants. MPC enables privacy-preserving machine learning applications such as oblivious inference. To see this, consider the two-party case with *f*(*M*, *x*) = *M*(*x*), where *M* and *x* are respectively the private machine learning model and data owned by the two parties.

ZKP was introduced by Goldwassor *et al.* [2] also in the 1980s. A ZKP system involves two participants: a prover and a verifier. The prover convinces the verifier that the secret information in his possession satisfies a certain NP relation, without revealing any information beyond the validity of the relation. As an important cryptographic primitive, ZKP also has potential applications in machine learning. For instance, in a bug-bounty program, people get rewarded for finding vulnerabilities of a publicly available pre-trained model *M*. Using ZKP, people can prove their knowledge of the bug without revealing it, e.g. the existence of two ‘close’ inputs *x*_1_ and *x*_2_ that yield different predictions *M*(*x*_1_) ≠ *M*(*x*_2_).

FHE was introduced by Rivest *et al.* [3] in 1978 as an advanced form of encryption that permits computations over encrypted data. This yields the desired result in encrypted form that, when decrypted, corresponds to the computations performed on the unencrypted data. Depending on the operations supported, e.g. encryption schemes are additive/multiplicative or fully homomorphic if they support addition/multiplication or both operations, respectively. It was not until 2009, 30 years after the concept of FHE was introduced, that Gentry gave the first explicit construction. If made practical, FHE has many important applications in privacy-preserving outsourced computation. Resource-constrained clients, such as mobile phones or IoT devices, encrypt their private data with (fully) homomorphic encryption and upload them to a server in the cloud, which performs all the operations (e.g. model training) on ciphertexts, and returns the encrypted result to the client. The client decrypts to obtain the result while keeping it from the server.

The most efficient constructions of MPC, ZKP and HE are based on number theoretic assumptions such as the discrete logarithm and factoring. However, they are not quantum resistant due to Shor’s algorithm. In response, the United States, China and the EU countries have begun or are about to start the process of soliciting and standardizing post-quantum public-key cryptographic algorithms.

Quantum algorithms do not seem to have universal exponential speedups on all computationally hard problems. There are problems that do not succumb to quantum computers, including symmetric-key primitives (e.g. hash functions and block ciphers), decoding problems (learning parity with noise, abbreviated as LPN) and lattice-related problems (e.g. learning with errors, abbreviated as LWE). When migrating the privacy-preserving technologies to the quantum-resistant version, we need to build the technologies upon these problems.

In terms of achieving post-quantum security for MPC, unconditionally secure (or information-theoretically secure) MPC protocols in the honest majority setting can trivially be made quantum safe. It seems that making a protocol information-theoretically secure is the simplest way to prevent quantum attacks. Actually, there are many information-theoretically secure MPC protocols dedicated to applications in machine learning, which enjoy better performance than computationally secure ones. However, these protocols have some limits such as the number of malicious parties cannot exceed half or one-third of the total number. There are even impossible results ruling out information-theoretically secure constructions in many settings. These theoretical results tell us that it is actually much harder to construct information-theoretically secure protocols.

The garbled circuit protocol of Yao [4] and the GMW protocol [5] are widely used in machine learning applications. Yao’s protocol involves a garbled circuit and oblivious transfer, while the GMW protocol uses only oblivious transfer. The garbled circuit method is typically instantiated with block ciphers (e.g. AES) or cryptographic hash functions (e.g. SHA-3), which can be made quantum resistant using the doubled key length or output length to counteract the quadratic speedup of the Grover algorithm. In principle, oblivious transfer can be based on (ring-)LWE, but the performance is not satisfactory for real applications. Recently, progress (e.g. [6]) has been made on building computation and communication efficient oblivious transfer protocols from variants of (ring-)LPN. These constructions significantly improve the efficiency of MPC protocols that heavily rely on oblivious transfer while preserving quantum resistance.

Additively homomorphic encryption is one of the widely used building blocks to construct MPC protocols. For example, random Beaver triples can be generated using additively homomorphic encryption schemes to securely compute multiplication (over binary or larger fields). The Paillier encryption is the most used candidate for additively homomorphic encryption, which is based on the hardness of factoring and is thus insecure against quantum computers. A promising alternative for post-quantum (additively) homomorphic encryption is to construct the encryption scheme based on (ring-)LWE.

MPC protocols are always run in a distributed framework, and authenticated broadcast channels are required to achieve the underlying security. It is also important to preserve the post-quantum security of these channels, but this is an independent topic of privacy-preserving computation; we omit detailed discussions in this article.

The research of general-purpose ZKP schemes has received renewed interest recently, especially due to the applications of zero-knowledge succinct non-interactive argument of knowledge (zk-SNARK) in blockchains. Most efficient zk-SNARK systems are based on elliptic-curve cryptography (ECC). These systems enjoy short proof size and fast verification time, and can be applied to prove the correctness of prediction and accuracy of machine learning models. However, the proving time is relatively large, and the memory consumption in the proving procedure is prohibitively huge for complex models. Furthermore, all these ECC-based schemes are not quantum resistant.

In contrast, ZKP systems based on symmetric ciphers such as the ‘MPC-in-the-head’ paradigm [7] and zero-knowledge scalable transparent arguments of knowledge (zk-STARK) [8] are quantum secure, but they are less supportive of complex statements like the inference of deep neural network models. Another line of ZKP systems stems from the recent progress in vector oblivious linear evaluation, e.g. [9,10], which is in turn based on the hardness of (ring-)LPN and symmetric ciphers. Although these ZKP systems have relatively long proof size and verification time, they are capable of proving very deep neural network models in practice.

We point out that, although zero-knowledge proof is a special case of two-party computation, it is also discussed in parallel because of the differences of underlying cryptographic tools and applications in privacy-preserving computation.

As for FHE, since the ground-breaking work of Gentry [11], significant progress has been made in the past decade to improve the concrete performance of FHE schemes, especially applications in machine learning training and inference. Almost all existing FHE constructions are based on (ring-)LWE and its variants, which are naturally quantum safe. In the applications of FHE in machine learning, the CKKS scheme [12] is commonly used to deal with arithmetic operations of floating-point numbers. Despite the desirable efficiency of CKKS in homomorphic arithmetic operations, it suffers from slow bootstrapping, which is the key procedure to support arbitrary homomorphic operations. Alternatives such as TFHE [13] and FHEW [14] have relatively faster bootstrapping procedures, but they are dedicated to bit operations. Since complex machine learning models consist of both linear and non-linear layers, it is of significant practical value to combine these two types of fully homomorphic encryption schemes to evaluate complex machine learning models on ciphertexts. Overall, the concrete performance of existing FHE schemes still remains far from the requirements of the applications in machine learning with complex models. It is a promising direction to accelerate the FHE schemes with hardware (e.g. GPU/FPGA).

It is worth pointing out that, although in principle quantum computers solve number-theoretic problems in polynomial time, the current state-of-the-art quantum computers are still far from breaking concrete crypto-systems such as RSA-1024. Despite the sustained progress of building universal quantum computers, we believe it leaves us sufficient time to migrate the existing cryptographic systems to their post-quantum versions. More specifically, (conjectured) quantum hard problems such as LPN and LWE constitute good candidates for building efficient and quantum resistant ZKP/MPC and FHE schemes, which ensure a smooth transition to the post-quantum era.

***Conflict of interest statement.*** None declared.
